# Loss of Pde6a Induces Rod Outer Segment Shrinkage and Visual Alterations in *pde6a^Q70X^* Mutant Zebrafish, a Relevant Model of Retinal Dystrophy

**DOI:** 10.3389/fcell.2021.675517

**Published:** 2021-05-20

**Authors:** Lucie Crouzier, Camille Diez, Elodie M. Richard, Nicolas Cubedo, Clément Barbereau, Mireille Rossel, Thomas Delaunay, Tangui Maurice, Benjamin Delprat

**Affiliations:** ^1^MMDN, Univ Montpellier, EPHE, INSERM, Montpellier, France; ^2^IES, Univ Montpellier, CNRS, Montpellier, France

**Keywords:** PDE6A, retinitis pigmentosa, photoreceptors, rods, cones, zebrafish

## Abstract

Retinitis pigmentosa (RP) is one of the most common forms of inherited retinal degeneration with 1/4,000 people being affected. The vision alteration primarily begins with rod photoreceptor degeneration, then the degenerative process continues with cone photoreceptor death. Variants in 71 genes have been linked to RP. One of these genes, *PDE6a* is responsible for RP43. To date no treatment is available and patients suffer from pronounced visual impairment in early childhood. We used the novel zebrafish *pde6a^Q70X^* mutant, generated by N-ethyl-N-nitrosourea at the European Zebrafish Resource Centre, to better understand how PDE6a loss of function leads to photoreceptor alteration. Interestingly, zebrafish *pde6a^Q70X^* mutants exhibited impaired visual function at 5 dpf as evidenced by the decrease in their visual motor response (VMR) compared to *pde6a*^*WT*^ larvae. This impaired visual function progressed with time and was more severe at 21 dpf. These modifications were associated with an alteration of rod outer segment length at 5 and 21 dpf. In summary, these findings suggest that rod outer segment shrinkage due to Pde6a deficiency begins very early in zebrafish, progresses with time. The zebrafish *pde6a^Q70X^* mutant represents an ideal model of RP to screen relevant active small molecules that will block the progression of the disease.

## Introduction

Ocular diseases, such as photoreceptor dystrophy, *retinitis pigmentosa* (RP), glaucoma, cataract or corneal dystrophy, lead to visual impairment. The degeneration of photoreceptors is one of the most common causes of hereditary blindness. In vertebrates, there are two classes of photoreceptors: the rods, responsible for dark vision, and the cones, responding to a bright light and giving colors vision (Fu, 1995; Kawamura and Tachibanaki, 2008). RP can be inherited in an autosomal recessive, dominant and X-linked manner. RP is a progressive disorder, characterized by the initial degeneration of rods followed by a secondary degeneration of cones (Tsang and Sharma, 2018a, b; Verbakel et al., 2018). Variants in 71 different genes^1^, have been associated with RP so far, among which *PDE6a* gene encodes the alpha subunit of cyclic phosphodiesterase 6-GMP (cGMP), expressed in the rods of the retina (Dryja et al., 1995; Huang et al., 1995).

Light detection by rod and cone photoreceptors of vertebrate retina utilizes cGMP as an intracellular messenger in the conversion of photon stimulation into an electrical response. Cyclic nucleotide phosphodiesterases (PDEs) catalyze the hydrolysis of 3′, 5′-cyclic nucleotides into inactive 5′-monophosphates. Five of the 11 PDE families contain regulatory segments called GAF-domain (GAF A and GAF B) that are located N-terminally to the more conserved catalytic domains (Aravind and Ponting, 1997). The GAF domain is named after some of the proteins it is found in, including cGMP-specific phosphodiesterases, adenylyl cyclases and FhlA. It is also present in guanylyl cyclases and phytochromes (Aravind and Ponting, 1997; Heikaus et al., 2009). Adenylyl and guanylyl cyclases catalyze ATP and GTP to the second messengers cAMP and cGMP, respectively. These products up-regulate catalytic activity by binding to the regulatory GAF domain(s). The opposite hydrolysis reaction is catalyzed by phosphodiesterase. cGMP-dependent 3′,5′-cyclic phosphodiesterase catalyzes the conversion of guanosine 3′,5′-cyclic phosphate to guanosine 5′-phosphate (Corbin et al., 2000). cGMP regulates catalytic activity by GAF-domain binding (Gillespie and Beavo, 1989) in the photoreceptor PDE6 family and provides one mechanism for regulating visual signal transduction.

PDE6a protein is therefore an effector enzyme of phototransduction and participates in the processes of transmission and amplification of the visual signal. Previous studies have revealed that variants of *PDE6a* gene lead to the degeneration of rods (Sakamoto et al., 2009; Sothilingam et al., 2015; Takahashi et al., 2018).

RP causes a gradual loss of vision which can extend over several years and today no effective treatment alleviates retinal disorders. It is therefore urgent to identify new therapeutic molecules that can slow the progression of the disease or preserve the vision in order to improve the patients’ quality of life. For this purpose, zebrafish is a model of choice as 70% of human genes have a homolog in zebrafish and 84% of its genes are associated with human pathologies (Howe et al., 2013). Several procedures have been set up to allow the high-throughput screening of behavioral alterations in zebrafish larvae. We therefore characterized a mutant zebrafish line expressing *pde6a^Q70X^* as a study model for the development of therapies to treat photoreceptor degeneration in humans, particularly suitable for *in vivo* screening. We here detail its morphological and behavioral phenotyping.

## Materials and Methods

### Zebrafish Generation, Maintenance, and Breeding

*Pde6a^*sa*14581^* carrier zebrafish were created by N-ethyl-N-nitrosourea (ENU) mutagenesis in the AB/Tupfel long fin mixed strain and were purchased from the European Zebrafish Resource Centre (EZRC) (Karlsruhe, Germany). The mutation changed the glutamine in position 70 into a stop codon and has been named *pde6a^Q70X^*. Heterozygous carriers of the mutation were outcrossed three times against wild type (AB strain) at EZRC and were subsequently intercrossed two times in our laboratory. Heterozygous fish of the resulting offspring were then used to produce embryos. The heterozygous *pde6a^Q70X^* mutant line was maintained in an automated fish tank system (ZebTEC, Tecniplast) at 28°C, pH 7, conductivity around 500 μS and with a 14 h:10 h light:dark cycle. They were fed a standard diet. Heterozygous *pde6a^+/Q70X^* zebrafish were intercrossed to generate homozygous wild-type (*pde6a*^*WT*^) and mutant *pde6a* zebrafish (*pde6a^Q70X^*). These fish became breeders and were incrossed in order to generate future generations for the experimental procedures. Embryos were collected from natural spawning, raised at 28.5°C in E3 medium (5 mM NaCl, 0.17 mM KCl, 0.33 mM CaCl_2_, 0.33 mM MgSO_4_, 0.05% methylene blue). Unfertilized embryos were discarded, and the water was changed only the first day. Larvae were euthanized by an extended bath of ice for biochemical experiments and immobilized in a 2.5% methylcellulose solution for imaging. Each experimental procedure was carried out in triplicate and larvae were from three different crosses.

### Genotyping

For isolated homozygous *pde6a*^*WT*^ and *pde6a^Q70X^* zebrafish, genomic DNA was extracted from the tail fins at 2 months of age by lysing the tissue in 50 mM NaOH at 95°C during 1 h (Meeker et al., 2007). A fragment of the *pde6a* gene was amplified by PCR using GoTaq Green Master Mix (Promega, Madison, United States) and the following primers: *pde6a* forward = 5′-ATAGGACAACTGTGACCCCACA-3′; *pde6a* reverse = 3′-GTGACATCAGTACGTACGTTGACAG-5′. The PCR program included initial denaturing at 95°C for 5min, followed by 25 cycles of denaturation at 95°C for 30 s, annealing at 55°C for 30 s, and elongation at 72°C for 30 s. These primers amplify a 539-bp region encompassing the *pde6a^Q70X^* mutation site. The products were subsequently digested with restriction enzyme Mwo1 (Thermo Fisher Scientific, ER1732), which is able to cut the WT amplicon twice and the mutant only once as the mutation abolishes one Mwo1 specific restriction site. For analysis, the digested products were separated by electrophoresis on a 2% agarose gel. The WT digested amplicon should give three bands of 271, 121, and 148 bp while the mutant should give two bands of 392 and 148 bp. The mutation was confirmed by Sanger sequencing.

### RT-PCR and Quantitative Real-Time PCR (qPCR)

Twenty whole *pde6a*^*WT*^ or *pde6a^Q70X^* larvae were collected and pooled at 5 days post fertilization (dpf) and total RNAs were extracted using a Nucleospin RNA Kit (Macherey-Nagel, Hoerdt, France) according to the manufacturer’s instructions. RNAs were reverse transcribed using M-MLV reverse transcriptase and quantitative RT-PCR were performed on a LightCycler 480 system (Roche, Boulogne-Billancourt, France). Primer sequences of zebrafish genes *pde6a* or *zef1*α were as follows: *pde6a* forward = 5′-ATGGCAGTCAACAAGATCGG-3′; *pde6a* reverse: 3′-TCATGTCGAGAAGGCCAACA-5′; *zef1*α forward: 5′-TTCTGTTACCTGGCAAAGGG-3′; *zef1*α reverse: 3′-TTCAGTTTGTCCAACACCCA-5′.

Control reactions were conducted with sterile water to determine signal background and DNA contamination. The standard curve of each gene was confirmed to be in a linear range, while *zef1*α gene was selected as reference.

### Visual Motor Response (VMR) Assay

The VMR assay quantifies the locomotor activity of zebrafish larvae to light changes using infrared tracking system. At 4 dpf, individual larvae were transferred in a 96 well plate (Whatman, #7701-1651, square and flat bottom wells) with 300 μl embryo medium and at 3 weeks post fertilization, the individual zebrafish were transferred in wells of a 24 well plate (Falcon^®^, 353047, square and flat bottom wells) with 2 ml embryo medium. *pde6a*^*WT*^ larvae were deposited in even wells and mutants in odd wells. Larvae without swim bladders or presenting visible physical deformities were not used for the experiment.

The locomotor behavior was monitored with an automated videotracking device (Zebrabox^®^, ViewPoint, Lissieu, France), employing a DinionXF 1/3-inch Monochrome camera (model BFLY-PGE-13H2M, Flir, Oregon, United States) fitted with a fixed-angle megapixel lens (SR5014MP-5C, SEIKO Optical, Willich, Germany). In the device, larvae in the plate were isolated from environmental surrounding noise. The floor of the box emitted by a light-controlling unit a white light (69–83 μW/cm^2^ measured at 495 λ). The response to stimuli was recorded by an infra-red (IR) camera (25 frames/s) under IR light illumination at 850 nm, which the animals could not perceive.

To characterize the rod physiology, zebrafish were acclimatized in the dark (0% brightness) for 1 h without tracking, then followed the same protocol with light (1% brightness; 3.5 Lux) and dark (0% brightness). In addition, in order to further reduce the contrast of light changes, a neutral density filter 8 (ND8) was placed on the light source. This was repeated twice, giving a total experiment duration of 100 min. The brightness changes were immediate (<<1 s).

The detection sensitivity was set to 31, activity burst threshold to 30 and activity freeze threshold to 10. Locomotor activity between the two threshold values was considered as normal activity. The distance covered was measured in mm/s. The values obtained in OFF were subtracted for each larvae by their ON values in order to remove the difference in basic locomotion that one could have between groups.

### Optokinetic Response (OKR) Assay

Larvae were randomly chosen and placed per group of four in a Petri dish (35 mm diameter) containing 2 ml of 2.5% methylcellulose (#9004-65-3, Sigma Aldrich, Missouri, United States). Using a tip, larvae were placed dorsal up and forming an X to avoid touching and interfering with each other. All measurements were done in the afternoon between 2:00 p.m. and 6:00 p.m. The room temperature was 28°C and the light was off.

Visual system performance of larval zebrafish was assessed using a videotracking device (VisioBox^®^, ViewPoint). Forty 6 mm-wide black and white strips were projected at a speed of 2 rpm on a white cylinder surrounding the fish (9 cm diameter). Larvae were trained for 1 min to the movement of the bands, going from left to right. Eye saccades were videotracked for 1 min. The direction of rotation of the bands was then reversed. The fish were trained again during 1 min and finally videotracked for 1 min.

The IR illuminated larvae from the bottom and responses were tracked using a FL3-U3-32S2M 1/2.8-inch Monochrome camera (FLEA3, Flir) at 25 frames/s. The macrolens had a focus at 0.10x (VS-MCA10, VS Technology, Tokyo, Japan). The number of eye saccades was manually measured (PHIVisualize software) and the average number of saccades per 2 min quantified.

### Morphological Analyses

Images were obtained using a stereo microscope (Olympus, Tokyo, Japan) equipped with a digital camera. Larvae were placed in a petri dish containing 2.5% methylcellulose. The total body length and eye diameter of the larvae were measured on an image taken at ×3.2 magnification. Ear area and anterior and posterior otoliths were measured at ×6.3 magnification. Images were processed with the ImageJ v1.46 software (NIH, Bethesda, MD, United States) to perform the various measurements.

### Photoreceptors Counting

Images were taken in the same session to ensure identical microscope settings used within the different groups. Individual cone cells, immunolabeled with Zpr-1 antibody, were quantified from three sections per fish (*n* = 14 for *pde6a*^*WT*^ and *n* = 12 for *pde6a^Q70X^* for 5 dpf larvae; *n* = 14 for *pde6a*^*WT*^ and *n* = 13 for *pde6a^Q70X^* for 21 dpf zebrafish). The total area of photoreceptor outer segments (immunolabeled with Rho4D2 antibody, three sections per fish, *n* = 3 for *pde6a*^*WT*^ and *n* = 3 for *pde6a^Q70X^* for 5 dpf larvae; *n* = 3 for *pde6a*^*WT*^ and *n* = 3 for *pde6a^Q70X^* for 21 dpf zebrafish) was measured using ImageJ, by selecting each region of interest and utilizing the Measurement tool. Average active caspase-3 positive cell number was measured by counting the number of positive cells (immunolabeled with a cleaved caspase-3 antibody) from three sections per fish (*n* = 16 for *pde6a*^*WT*^ and *n* = 15 for *pde6a^Q70X^* for 5 dpf larvae; *n* = 10 for *pde6a*^*WT*^ and *n* = 10 for *pde6a^Q70X^* for 21 dpf zebrafish). Individual cone numbers and total area of rod outer segments were normalized against the total length of the retina in μm. The number of active Caspase-3 positive cells was normalized per whole retinal section.

### Immunohistochemistry and Confocal Microscopy

Larvae were fixed in 4% paraformaldehyde for 24 h at 4°C and dehydrated in 25% sucrose-PBS for 24 h then in 35% sucrose-PBS for 24 h. Embryos were placed in plastic molds, oriented in Cryomatrix (O.C.T.^TM^, Sakura, Tissue-Tek, Netherlands) at room temperature and rapidly frozen in liquid nitrogen.

Larvae were transversely sectioned in 10 μm-thick slices, using a cryostat (Leica, Wetzlar, Germany) at –20°C and mounted on positively charged glass slides. To prevent unspecific binding of the antibody, cryosections were blocked with 0.1% PBS/Triton X-100, 5% Horse serum for 30min at room temperature and incubated overnight at 4°C with either an anti-Rho4d2 antibody (dilution 1:7000; ab98887, Abcam, Cambridge, UK), an anti-ZPR-1 antibody (dilution 1:500; ab174435, Abcam) or an anti-cleaved Caspase-3 (Asp175) antibody (dilution 1:500, #9661, Cell Signaling Technology, United States). The sections were incubated 2 h at room temperature with the following secondary antibodies conjugated with Cy3 anti-mouse (dilution 1:800; 715-165-150, Jackson ImmunoResearch), Cy3 anti-rabbit (dilution 1:1,000; 711-166-152, Jackson ImmunoResearch) or Alexa Fluor^®^ 488 anti-mouse (dilution 1:1,000; 715-545-150, Jackson ImmunoResearch). Nuclei were counterstained with 4′,6-diamidino-2-phenylendole (DAPI; dilution 1:5,000; Sigma Aldrich). The emitted fluorescence was measured using a confocal microscope (LSM880 Fastairyscan, Zeiss).

### Statistical Analyses

Statistical analysis was performed using Prism^TM^ v5.0 software (GraphPad, San Diego, CA, United States). To compare two experimental groups *(pde6a^*WT*^* vs. *pde6a^Q70X^*), one-tailed Student’s *t*-test was used. All data were presented as mean ± standard deviation (SD). The levels of statistical significance were: ^∗^*p* < 0.05, ^∗∗^*p* < 0.01, ^∗∗∗^*p* < 0.001, and ^****^p < 0.0001.

## Results

### Generation of a *pde6a^Q70X^* Mutant Zebrafish Line

**FIGURE 1:**
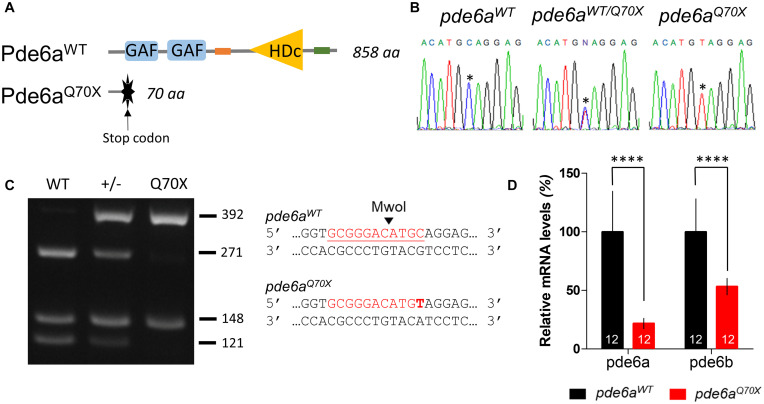
Mutation in *pde6a* zebrafish causes a premature stop codon. **(A)** Structure of zebrafish Pde6a protein with specific GAF (cGMP-specific phosphodiesterases, adenylyl cyclases and FhlA) and HDc [histidine (H) and/or aspartate (D) amino acid residues] domains. **(B)** Sanger sequencing identified the nucleotide mutation in exon 1 of *pde6a* sequence. Asterisk (*) denotes the substituted nucleotide in the knock-out *pde6a* sequencing trace. **(C)** A targeted fragment was amplified by PCR from the genomic DNA of adult zebrafish tail followed by an enzymatic digestion with Mwo1. The three rows present digested products for *pde6a^Q70X^*, *pde6a*^*WT*^, and *pde6a^*WT*/Q70X^* fish. Mwo1 restriction site is shown on the side of the agarose gel image. The mutation is highlighted in bold. **(D)** Expression level of *pde6a* and *pde6b* mRNA revealed by qPCR in *pde6a^Q70X^* and *pde6a*^*WT*^ zebrafish larvae. Expression of *pde6a* and *pde6b* were normalized using the *zef1*α reference gene. Error bars represent SD calculated from three replicas. ^****^p < 0.0001.

To determine the role of Pde6a in photoreceptor function, we analyzed *pde6a^Q70X^* mutant zebrafish, generated using the ENU method, at the EZRC. The mutation results in the production of a truncated protein (less than 10% of the wild type protein should be produced) and disrupts almost all of the functional domains (Figure 1A). Heterozygous *pde6a^+/Q70X^* zebrafish were intercrossed to generate *pde6a*^*WT*^, *pde6a^+/Q70X^*, and *pde6a^Q70X^* littermates. Sanger sequencing of several larvae genomic DNA confirmed the mutation, with wild type allele (C) in *pde6a*^*WT*^ and the ENU mutant allele (T) in *pde6a^Q70X^*. The heterozygous fish showed the wild type (C) and ENU mutant alleles (T) (Figure 1B). The genotypes of the fish were assessed by PCR and Mwo1 endonuclease digestion, as the recognition of the restriction site is disrupted by the *pde6a^Q70X^* mutation. As expected, *pde6a*^*WT*^ digested amplicon resulted in three distinct bands migrating at 121, 148, and 271 bp, while the *pde6a^Q70X^* digested amplicon showed two bands at 148 and 392 bp (Figure 1C). Finally, in order to determine if the mutation induced mRNA decay, qPCR experiment was achieved. qPCR showed that larvae carrying the *pde6a^Q70X^* mutation had decreased *pde6a* expression level compared to controls (Figure 1D). Curiously, *pde6b* expression level was also decreased in the *pde6a^Q70X^* mutant. Thus, these results confirmed that a mutant zebrafish line deficient in pde6a had indeed been generated.

### Zebrafish *pde6a^Q70X^* Mutants Do Not Develop Gross Morphological Alteration

**FIGURE 2:**
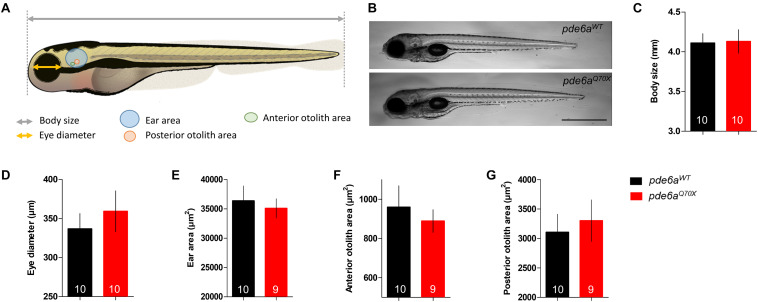
Morphology of the *pde6a^Q70X^* larvae at 5-dpf. **(A)** Schematic representation of the different measurements of the larva (modified from Lizzy Griffiths). **(B)** *pde6a^Q70X^* larvae showed presence of swim bladders and absence of any morphological abnormality as compared to *pde6a*^*WT*^ controls. **(C)** Measurement of body length, from the mouth to the end of the tail fin and **(D)** the diameter of the eye. The area of the ear **(E)**, anterior **(F)**, and posterior **(G)** otolith were also analyzed. Scale = 1 mm. The number of animals is indicated within the columns. *p* > 0.05 for all comparisons.

Genotyping of numerous larvae from *pde6a^+/Q70X^* intercrosses revealed that homozygous offspring (*pde6a^Q70X^*) appeared normal and were present in Mendelian ratios at 5 dpf. To decipher the impact of *pde6a* gene mutation on zebrafish development, morphological analyzes were carried out at the same stage than behavioral tests: 5 dpf. The body length, eye diameter, ear area as well as posterior and anterior otolith area were measured (Figure 2A). Neither the body length (Figures 2B,C), nor the eye diameter were different between *pde6a*^*WT*^ and *pde6a^Q70X^* (Figure 2D). In addition, no significant difference was found in ear area (Figure 2E), or anterior (Figure 2F) and posterior (Figure 2G) otolith areas. Taken together, these data suggested that the disruption of Pde6a level did not induce gross morphological alterations in *pde6a^Q70X^* line.

### Zebrafish *pde6a^Q70X^* Mutants Have Impaired Visual Function

**FIGURE 3:**
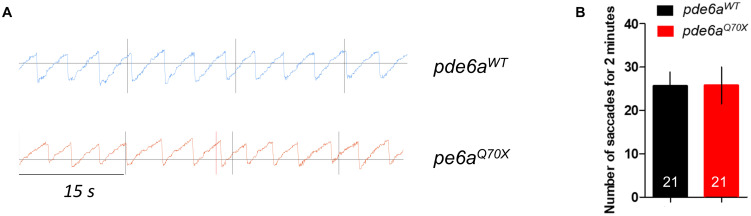
Impact of *pde6a* mutation on the OKR test of larvae at 5-dpf. **(A)** OKR chromograph illustrating the saccades performed by *pde6a*^*WT*^ larvae (in blue) and *pde6a^Q70X^* larvae (in red) for 1 min. **(B)** Quantification of the number of saccades performed in 2 min. The number of animals is indicated within the columns. The experiment was repeated 3 times.

Visual function was first assessed in *pde6a^Q70X^* mutant larvae and control siblings at 5 dpf using the optokinetic response (OKR). In the OKR test, saccade movements of larvae in response to black and white stripes were measured (Figure 3A), reflecting the visual acuity of the larvae. No difference in the number of saccades was observed between p*de6a*^*WT*^ and *pde6a^Q70X^* larvae (Figures 3A,B). The decrease of Pde6a level did not impair the visual acuity of the larvae at 5 dpf.

**FIGURE 4:**
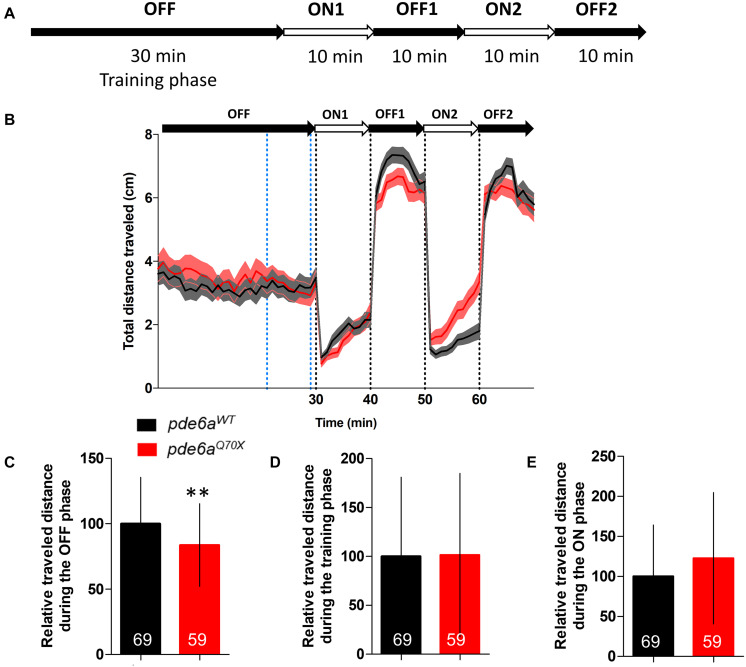
Analysis of the distance traveled by the larvae during the light/dark cycle in the VMR test at 5 dpf. **(A)** Sequence protocol: the activity is measured for 70 min, with 30 min of training in the dark (OFF), then 2 cycles of light/dark (ON/OFF) of 10 min each. **(B)** Average distance traveled per min for each condition according to the light/dark protocol. Distance traveled by the larvae during: **(C)** the averaged OFF1 and OFF2 phases; **(D)** the training phase [blue dotted lines in **(B)**, between 21 and 29 min]; and **(E)** the averaged ON1 and ON2 phases. Distance were expressed as % of controls. Error bars represent the SD from three replicas except for B where errors bars represent the SEM. The number of animals is indicated within the columns. ^∗∗^*p* < 0.01.

Then, we analyzed rod function using a modified protocol of the visual motor response (VMR). The initial VMR test measures locomotor activity of larvae in response to drastic changes from 100% brightness to 0% brightness, this change in brightness being presented twice sequentially. The light intensity of the 100% brightness activates the cones and will not reflect rod function. Consequently, we applied a neutral density filter 8 and changed the light intensity from 1 to 0% (Figure 4A). The locomotor response of the 5 dpf *pde6a^Q70X^* mutant larvae was significantly decreased in the OFF phases (Figures 4B,C). No difference in traveled distance was observed during the training (Figure 4D) and ON phases (Figure 4E).

**FIGURE 5:**
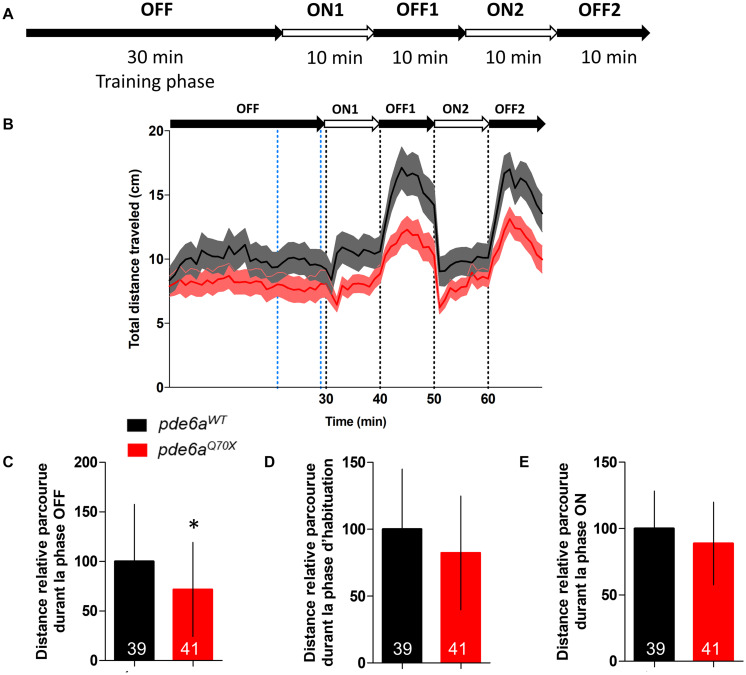
Analysis of the distance traveled by the larvae during the light/dark cycle in the VMR test at 21 dpf. **(A)** Sequence protocol: the activity is measured for 70 min, with 30 min of training in the dark (OFF), then 2 cycles of light/dark (ON/OFF) of 10 min each. **(B)** Average distance traveled per min for each condition according to the light/dark protocol. Distance traveled by the larvae during: **(C)** the averaged OFF1 and OFF2 phases; **(D)** the training phase [blue dotted lines in **(B)**, between 21 and 29 min]; and **(E)** the averaged ON1 and ON2 phases. Distance were expressed as % of controls. Error bars represent the SD from three replicas except for B where errors bars represent the SEM. The number of animals is indicated within the columns. ^∗^*p* < 0.05.

Since RP is a progressive retinal pathology, we analyzed the VMR of 21 dpf zebrafish. Using the same protocol (Figure 5A), a statistically significant decrease of the traveled distance was also observed between *pde6a*^*WT*^ and *pde6a^Q70X^* larvae in the OFF periods (Figures 5B,C). Similarly to the results obtained on 5dpf larvae, no difference in traveled distance was observed during the training (Figure 5D) and ON phases (Figure 5E) in 21 dpf larvae.

### Rod Photoreceptor Outer Segments Shrink in *pde6a^Q70X^* Mutants

**FIGURE 6:**
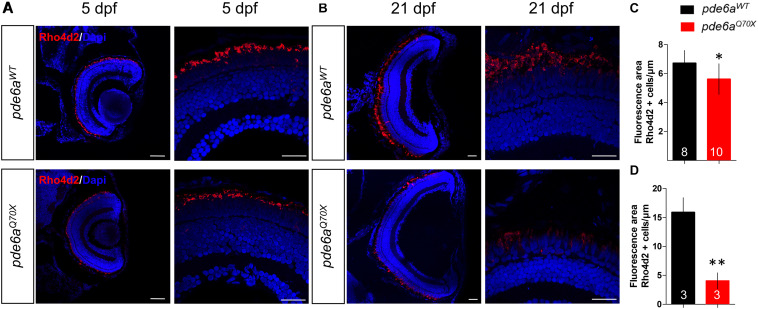
Analysis of rods in *pde6a^Q70X^* zebrafish during development. Representative confocal images of the retina of *pde6a*^*WT*^ and *pde6a^Q70X^* zebrafish obtained after immunostaining of rods with a Rho4d2 antibody highlighting Rhodopsin (red) at 5 dpf **(A)** and 21 dpf **(B)**. The nuclei were counterstained with DAPI (blue). Scale bar = 30 μm. **(C,D)** Quantification of the total area of rod outer segments normalized against the length of the retinal section, based on three independent measurements of larvae per group at 5 dpf **(C)** and 21 dpf **(D)**. The number of animals is indicated within the columns. ^∗^*p* < 0.05, ^∗∗^*p* < 0.01.

In order to determine if these behavioral deficits are correlated to histological alterations, we performed a histological analysis of the retina in *pde6a*^*WT*^ and *pde6a^Q70X^* larvae. A specific immunolabeling of Rhodopsin, with an anti-Rho4d2 antibody, was used to count and compare the number of rods between the two genotypes. At 5 dpf, the total area occupied by rod photoreceptor outer segments was significantly smaller in *pde6a^Q70X^* (5.61 μm^2^/μm of retina section ± 0.33) compared to *pde6a*^*WT*^ (6.71 μm^2^/μm ± 0.29) (Figures 6A,C). At 21 dpf, the total area occupied by rod photoreceptor outer segments was significantly smaller in *pde6a^Q70X^* (4.07 μm^2^/μm ± 0.79) compared to *pde6a*^*WT*^ (15.90 μm^2^/μm ± 1.47) (Figures 6B,D).

### Cone Photoreceptor Number Is Slightly Decreased in *pde6a^Q70X^* Mutants

**FIGURE 7:**
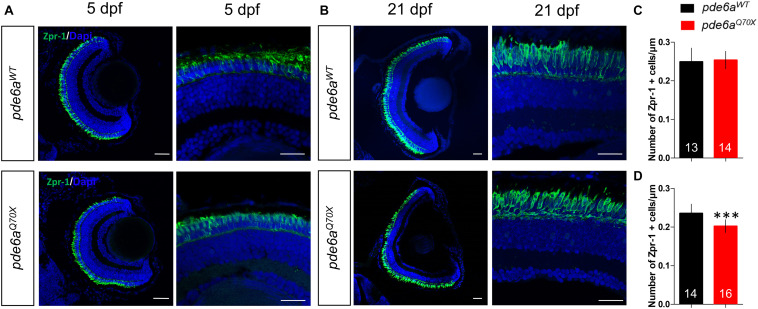
Analysis of cones in *pde6a^Q70X^* zebrafish during development. Representative confocal images of the retina of *pde6a*^*WT*^ and *pde6a^Q70X^* zebrafish obtained after immunostaining of cones with a Zpr-1 antibody (green) at 5 dpf **(A)** and 21 dpf **(B)**. Nuclei were highlighted with DAPI (blue). Scale bar = 30 μm. **(C,D)** Quantification of the number of cones normalized against the length of the retinal section, based on three independent measurements of larvae per group at 5 dpf **(C)** and 21 dpf **(D)**. The number of animals is indicated within the columns. ^∗∗∗^*p* < 0.001.

Since RP is a rod-cone dystrophy, the degeneration of the rods is supposed to be followed by cone degeneration. To check this hypothesis, we analyzed cone presence using the cone specific antibody Zpr-1. At 5 dpf, the number of cones present in the retina of *pde6a^Q70X^* larvae (0.25 cone/μm of retina section ± 0.006) was unchanged as compared to *pde6a*^*WT*^ (0.24 ± 0.009) (Figures 7A,C). However, at 21 dpf the number of cones of the *pde6a^Q70X^* larvae decreased significantly as compared to *pde6a*^*WT*^ (0.20 Zpr-1 positive cell/μm ± 0.004 vs. 0.23 Zpr-1 positive cell/μm ± 0.006, *p* < 0.001) (Figures 7B,D).

### Photoreceptors Degenerate by Apoptosis in *pde6a^Q70X^* Mutants

**FIGURE 8:**
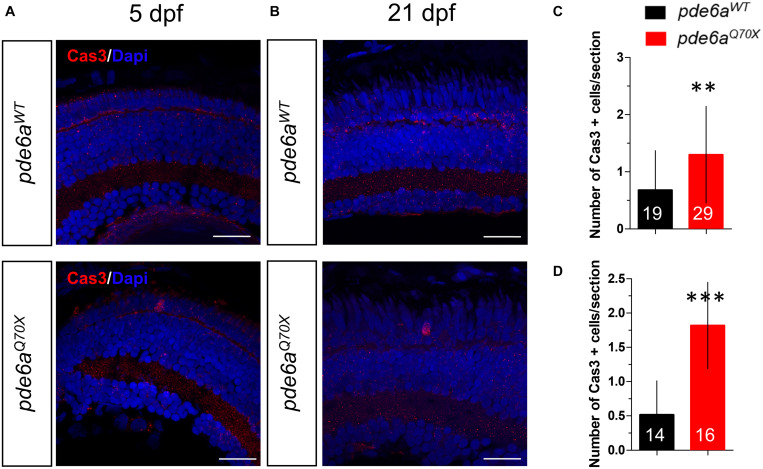
Analysis of activated Caspase-3 positive cells in *pde6a^Q70X^* zebrafish during development. **(A,B)** Representative confocal images of the retina of *pde6a*^*WT*^ and *pde6a^Q70X^* zebrafish obtained after immunostaining of the retinal section with activated Caspase-3 antibody (red) at 5 dpf **(A)** and 21 dpf **(B)**. Nuclei were highlighted with DAPI (blue). Scale bar = 30 μm. **(C,D)** Quantification of the number of activated Caspase-3 positive cells in the entire retina based on three independent measurements of larvae per group at 5 dpf **(C)** and 21 dpf **(D)**. The number of animals is indicated within the columns. ** *p* < 0.01, ***p < 0.001.

As photoreceptor degeneration is mainly due to apoptosis, we used a specific antibody that recognized activated Caspase-3 to evaluate the apoptotic cell death in the retina. Indeed, this protease enzyme is activated in the apoptotic cell by both extrinsic (death ligand) and intrinsic (mitochondrial) pathways (Salvesen, 2002). At 5 dpf, the number of cleaved-Caspase-3 positive cells present in the retina was higher in *pde6^*Q*70X^* (1.3 Caspase-3 positive cell/retinal section ± 0.15) compared to *pde6a*^*WT*^ (0.67 ± 0.15 Caspase-3 positive cell/retinal section) (Figures 8A,C) in the outer nuclear layer (ONL). This number was accentuated with development and was much higher in *pde6^*Q*70X^* (1.8 Caspase-3 positive cell/retinal section ± 0.19) compared to *pde6a*^*WT*^ (0.51 Caspase-3 positive cell/retinal section ± 0.14) at 21 dpf (Figures 8B,D) in the ONL.

## Discussion

The aim of this study was to generate and characterize a new zebrafish model of RP, the *pde6a^Q70X^* line. *pde6a* gene, encoding the alpha subunit of cyclic phosphodiesterase 6-GMP (cGMP), is expressed in the retinal rods. Mutations in human induce RP (Dryja et al., 1995; Huang et al., 1995; Khan et al., 2015; Sothilingam et al., 2015; Ullah et al., 2016). This pathology, characterized by night blindness developing during childhood, is due to photoreceptor degeneration (Huang et al., 1995). In mice, mutation of *Pde6a* leads to a rapid degeneration of rod photoreceptors followed by cone degeneration (Sakamoto et al., 2009). Interestingly, two mouse models were analyzed with different mutations in *Pde6a*. The pace of the degeneration varied, depending on the mutation *Pde6a^*nmf*282/nmf282^* (V685M) or *Pde6a^*nmf*363/nmf363^* (D670G). Indeed, the V685M lead to an earlier and faster photoreceptor degeneration than the one observed in mice harboring the D670G mutation, suggesting that some amino acids are more important for the function of the protein. Since the majority of the patients present with compound heterozygous mutations, Sothilingam et al. (2015) compared the severity of the phenotypes between three disease-causing variants, R562W, D670G, and V685M. They concluded that the severity depends on the mutation, confirming previous observations (Sakamoto et al., 2009). Finally, a mutation of *Pde6a* was identified in dog (Petersen-Jones et al., 1999). The sequence of the canine genomic DNA showed a 1-bp deletion in codon 616 of *Pde6a*, resulting in a frame shift leading to a stretch of 28 altered amino acids followed by a premature stop codon (Petersen-Jones et al., 1999). The dogs developed a massive photoreceptors degeneration as early as 5 weeks-old (Tuntivanich et al., 2009). By the age of 9 weeks, few cone cells were present while all rods had degenerated. This degeneration is associated with a complete loss of rod function measured by white-light flicker response (Tuntivanich et al., 2009).

Up to now, there is no treatment available to block the progression of the photoreceptor degeneration. Ongoing preclinical studies focused on the use of gene therapy. This approach was tested in mouse (Schon et al., 2017) and dog (Mowat et al., 2017; Occelli et al., 2017) but some adverse effects were described (Mowat et al., 2017). Altogether, these trials suggested that gene therapy may be used to correct the deficits observed in patients. However, these data should be taken with caution since the positive effect seemed limited to the site of injection. Another way to block or delay the progression of the photoreceptor degeneration in *Pde6a* patients may be the use of active molecules. The identification of good candidate(s) necessitates to screen huge batches of molecules. This method needs a relevant model that allows high throughput screening. In addition, to improve the efficacy of the molecules, *in vivo* models should be prioritized. One model recapitulates these two prerequisites, the zebrafish. Indeed, in recent years, zebrafish has emerged as an excellent model system for chemical screening (MacRae and Peterson, 2015; Rennekamp and Peterson, 2015; Brady et al., 2016). Zebrafish has been used to identify novel drugs or repurpose already used drugs in the indications of epilepsy, hearing loss, and cardiovascular system disorders (MacRae and Peterson, 2015). Very interestingly, zebrafish has become an extremely popular and useful animal model in the visual field (Ganzen et al., 2017). Indeed, zebrafish eye shares important similarities with the human eye. Notably, zebrafish have a canonical vertebrate retina composed of three nuclear layers, two plexiform layers, five neuronal cell types (photoreceptors, horizontal, bipolar, amacrine, ganglion cells) and one major glial cell type (Müller glia). Moreover, their retina contains two types of photoreceptors, rods and cones. Contrary to mouse models, zebrafish are, like human, diurnal. Interestingly, there is an increasing number of transgenic zebrafish models of retinal disease (Link and Collery, 2015). Finally, novel device allows the analysis of zebrafish vision in high-throughput screening using the VMR. The VMR is a vision-mediated startle response, initiated by a drastic change in illumination (Emran et al., 2008). In a typical VMR assay, larvae of 5 dpf are individually placed in a 96 multi-well plate placed inside a lightproof machine to isolate them from external disturbances and environmental light (Ganzen et al., 2017). Recently, this methodological approach has led to the identification of ocular antiangiogenic drugs (Ohnesorge et al., 2019).

In our study, we characterized a novel mutant zebrafish: *pde6a^Q70X^* which developed visual impairment as soon as 5 dpf as evidenced by our modified VMR experiments. These defects aggravated with age and were correlated with photoreceptor alteration. The loss of Pde6a could lead to rod degeneration or an alteration of their outer segments only. The decreased size of the area labeled with rhodopsin is not sufficient to have a clear answer. Nevertheless, since the number of apoptotic cells in the outer nuclear layer was modest, the most plausible explanation is that Pde6a deficiency leads to a reduced outer segment area rather than a drastic loss of rods at 21 dpf.

This modification was also associated with a slight loss of cones. Since the number of photoreceptors labeled with activated capase-3 is limited, it is challenging to determine if the decreased number of cones at 21 dpf is mainly due to apoptotic cell death or a reduction of cell differentiation in the retinal ciliary marginal zone. Moreover, although statistically significant, this loss is probably not sufficient to have a physiological relevance. Zebrafish vision is more driven by cones than rods due to the higher proportion of cones (60%) (Fadool, 2003; Angueyra and Kindt, 2018). Consequently, as *pde6a^Q70X^* zebrafish kept their ability to feed and lived normally until adult stage, the slight cone loss might only have a weak impact on vision, if any. In addition, since rod degeneration is not always followed by cone degeneration as observed when Rhodospin is mutated (Zelinka et al., 2018; Santhanam et al., 2020), or missorted (Morris et al., 2005), Pde6a deficiency may lead to rod outer segment shrinkage without gross alteration of cones at 21 dpf. Moreover, studies involving other key proteins such as IFT88, a cilium protein (Nakao et al., 2012), or toxic agents (Montgomery et al., 2010) did not report this sequential degeneration neither. In mammals, rods represent 90% of the photoreceptors and secrete RdCVF, a rod-derived cone viability factor (Ait-Ali et al., 2015). In contrast, in the zebrafish, rods are present in a lower proportion and their loss should have a limited impact on cone survival as hypothesized by Jaroszynska et al. (2021).

Function of rod photoreceptors in zebrafish larvae is still under debate but our data strongly support the important function of the rods in larval visual physiology. Our results are in accordance with the recent work by Venkatraman et al. (2020) who demonstrated the role of rods in larval visual activity. Another study demonstrated that VMR and OKR were altered, at 5dpf, in *slc7A14* knock-down zebrafish larvae, another model of RP (Zhuang et al., 2019). Interestingly, these deficits are supposed to be linked to an alteration of rods and retinal pigment epithelium (RPE), since no cone defects were observed. Nevertheless, we can’t exclude that the effect on OKR is due to defective RPE rather than defective rods. Finally, loss of Pde6a in our mutant zebrafish did not induce *Pde6b* overexpression, ruling out a potential rod preservation through a compensatory mechanism despite the recent demonstration that a pre-mature termination codon and a homologous sequence are required to elicit a gene compensation (Peng, 2019).

## Conclusion

In conclusion, the aim of this work was to generate and characterize a novel zebrafish model for *pde6a* mutations. The *pde6a^Q70X^* mutant zebrafish developed a visual defect as early as 5 dpf, that worsen with development. Our model could be used to efficiently screen active molecules that will block the alteration of the photoreceptors. Very interestingly, all molecules that will block this degeneration might be relevant to treat other form of RP such as Usher syndrome.

## Data Availability Statement

The raw data supporting the conclusions of this article will be made available by the authors, without undue reservation.

## Ethics Statement

Ethical review and approval were not required for the animal study because experiments were designed and performed according to EU directive 2010/63/UE and French application decree 2013-118. In particular, most of the experiments were done with 5 dpf larvae which are out of the scope of the regulation. Regarding P21 zebrafish, Art. R. 214-88 stipulates that practices which are likely to cause pain, suffering, anguish or lasting damage less than those caused by the introduction of a needle carried out in accordance with good veterinary practice does not need ethical committee authorization. Experiments consisted only in the observation of the spontaneous behavior of the animals and thus also were out of the scope of the regulation.

## Author Contributions

BD and TM conceived and designed experiments. LC, ER, CD, CB, TD and MR performed the experiments and analyzed the data. NC took care of the zebrafish. BD and LC wrote the manuscript. All authors contributed to the article and approved the submitted version.

## Conflict of Interest

The authors declare that the research was conducted in the absence of any commercial or financial relationships that could be construed as a potential conflict of interest.

## Footnotes

1 https://sph.uth.edu/retnet

## References

[B1] Ait-Ali N. Fridlich R. Millet-Puel G. Clerin E. Delalande F. Jaillard C. (2015). Rod-derived cone viability factor promotes cone survival by stimulating aerobic glycolysis. *Cell* 161 817–832. 10.1016/j.cell.2015.03.023 25957687

[B2] Angueyra J. M. Kindt K. S. (2018). Leveraging Zebrafish to study retinal degenerations. *Front. Cell Dev. Biol.* 6:110. 10.3389/fcell.2018.00110 30283779 PMC6156122

[B3] Aravind L. Ponting C. P. (1997). The GAF domain: an evolutionary link between diverse phototransducing proteins. *Trends Biochem. Sci.* 22 458–459. 10.1016/s0968-0004(97)01148-1 9433123

[B4] Brady C. A. Rennekamp A. J. Peterson R. T. (2016). Chemical screening in zebrafish. *Methods Mol. Biol.* 1451 3–16. 27464797 10.1007/978-1-4939-3771-4_1

[B5] Corbin J. D. Turko I. V. Beasley A. Francis S. H. (2000). Phosphorylation of phosphodiesterase-5 by cyclic nucleotide-dependent protein kinase alters its catalytic and allosteric cGMP-binding activities. *Eur. J. Biochem.* 267 2760–2767. 10.1046/j.1432-1327.2000.01297.x 10785399

[B6] Dryja T. P. Finn J. T. Peng Y. W. Mcgee T. L. Berson E. L. Yau K. W. (1995). Mutations in the gene encoding the alpha subunit of the rod cGMP-gated channel in autosomal recessive retinitis pigmentosa. *Proc. Natl. Acad. Sci. U.S.A.* 92 10177–10181. 7479749 10.1073/pnas.92.22.10177 PMC40759

[B7] Emran F. Rihel J. Dowling J. E. (2008). A behavioral assayto measure responsiveness of zebrafish to changes in light intensities. *J. Vis. Exp.* 20:923. 10.3791/923 19078942 PMC2879884

[B8] Fadool J. M. (2003). Rod genesis in the teleost retina as a model of neural stem cells. *Exp. Neurol.* 184 14–19. 10.1016/s0014-4886(03)00309-1 14637072

[B9] Fu Y. (1995). “Phototransduction in rods and cones,” in *Webvision: The Organization of the Retina and Visual System*, eds Kolb H. Fernandez E. Nelson R. (Salt Lake City, UT: University of Utah Health Sciences Center). 21413389

[B10] Ganzen L. Venkatraman P. Pang C. P. Leung Y. F. Zhang M. (2017). Utilizing zebrafish visual behaviors in drug screening for retinal degeneration. *Int. J. Mol. Sci.* 18:1185. 10.3390/ijms18061185 28574477 PMC5486008

[B11] Gillespie P. G. Beavo J. A. (1989). cGMP is tightly bound to bovine retinal rod phosphodiesterase. *Proc. Natl. Acad. Sci. U.S.A.* 86 4311–4315. 2542968 10.1073/pnas.86.11.4311 PMC287442

[B12] Heikaus C. C. Pandit J. Klevit R. E. (2009). Cyclic nucleotide binding GAF domains from phosphodiesterases: structural and mechanistic insights. *Structure* 17 1551–1557. 20004158 10.1016/j.str.2009.07.019 PMC2801740

[B13] Howe K. Clark M. D. Torroja C. F. Torrance J. Berthelot C. Muffato M. (2013). The zebrafish reference genome sequence and its relationship to the human genome. *Nature* 496 498–503. 23594743 10.1038/nature12111 PMC3703927

[B14] Huang S. H. Pittler S. J. Huang X. Oliveira L. Berson E. L. Dryja T. P. (1995). Autosomal recessive retinitis pigmentosa caused by mutations in the alpha subunit of rod cGMP phosphodiesterase. *Nat. Genet.* 11 468–471. 10.1038/ng1295-468 7493036

[B15] Jaroszynska N. Harding P. Moosajee M. (2021). Metabolism in the zebrafish retina. *J. Dev. Biol.* 9:10. 10.3390/jdb9010010 PMC8006245 33804189

[B16] Kawamura S. Tachibanaki S. (2008). Rod and cone photoreceptors: molecular basis of the difference in their physiology. *Comp. Biochem. Physiol. A Mol. Integr. Physiol.* 150 369–377. 18514002 10.1016/j.cbpa.2008.04.600

[B17] Khan S. Y. Ali S. Naeem M. A. Khan S. N. Husnain T. Butt N. H. (2015). Splice-site mutations identified in PDE6A responsible for retinitis pigmentosa in consanguineous Pakistani families. *Mol. Vis.* 21 871–882. 26321862 PMC4539017

[B18] Link B. A. Collery R. F. (2015). Zebrafish models of retinal disease. *Annu. Rev. Vis. Sci.* 1 125–153. 28532376 10.1146/annurev-vision-082114-035717

[B19] MacRae C. A. Peterson R. T. (2015). Zebrafish as tools for drug discovery. *Nat. Rev. Drug Discov.* 14 721–731. 26361349 10.1038/nrd4627

[B20] Meeker N. D. Hutchinson S. A. Ho L. Trede N. S. (2007). Method for isolation of PCR-ready genomic DNA from zebrafish tissues. *Biotechniques* 43 610,612,614. 10.2144/000112619 18072590

[B21] Montgomery J. E. Parsons M. J. Hyde D. R. (2010). A novel model of retinal ablation demonstrates that the extent of rod cell death regulates the origin of the regenerated zebrafish rod photoreceptors. *J. Comp. Neurol.* 518 800–814. 10.1002/cne.22243 20058308 PMC3656417

[B22] Morris A. C. Schroeter E. H. Bilotta J. Wong R. O. Fadool J. M. (2005). Cone survival despite rod degeneration in XOPS-mCFP transgenic zebrafish. *Invest. Ophthalmol. Vis. Sci.* 46 4762–4771. 10.1167/iovs.05-0797 16303977 PMC2810103

[B23] Mowat F. M. Occelli L. M. Bartoe J. T. Gervais K. J. Bruewer A. R. Querubin J. (2017). Gene therapy in a large animal model of PDE6A-retinitis pigmentosa. *Front. Neurosci.* 11:342. 10.3389/fnins.2017.00342 28676737 PMC5476745

[B24] Nakao T. Tsujikawa M. Notomi S. Ikeda Y. Nishida K. (2012). The role of mislocalized phototransduction in photoreceptor cell death of retinitis pigmentosa. *PLoS One* 7:e32472. 10.1371/journal.pone.0032472 22485131 PMC3317642

[B25] Occelli L. M. Schon C. Seeliger M. W. Biel M. Michalakis S. Petersen-Jones S. (2017). Gene supplementation rescues rod function and preserves photoreceptor and retinal morphology in dogs, leading the way towards treating human PDE6A-retinitis pigmentosa. *Hum. Gene Ther.* 28 1189–1201. 10.1089/hum.2017.155 29212382

[B26] Ohnesorge N. Sasore T. Hillary D. Alvarez Y. Carey M. Kennedy B. N. (2019). Orthogonal drug pooling enhances phenotype-based discovery of ocular antiangiogenic drugs in zebrafish larvae. *Front. Pharmacol.* 10:508. 10.3389/fphar.2019.00508 31178719 PMC6544088

[B27] Peng J. (2019). Gene redundancy and gene compensation: an updated view. *J. Genet. Genomics* 46 329–333. 10.1016/j.jgg.2019.07.001 31377237

[B28] Petersen-Jones S. M. Entz D. D. Sargan D. R. (1999). cGMP phosphodiesterase-alpha mutation causes progressive retinal atrophy in the Cardigan Welsh corgi dog. *Invest. Ophthalmol. Vis. Sci.* 40 1637– 1644. 10393029

[B29] Rennekamp A. J. Peterson R. T. (2015). 15 years of zebrafish chemical screening. *Curr. Opin. Chem. Biol.* 24 58–70. 25461724 10.1016/j.cbpa.2014.10.025 PMC4339096

[B30] Sakamoto K. Mccluskey M. Wensel T. G. Naggert J. K. Nishina P. M. (2009). New mouse models for recessive retinitis pigmentosa caused by mutations in the Pde6a gene. *Hum. Mol. Genet.* 18 178–192. 10.1093/hmg/ddn327 18849587 PMC2644649

[B31] Salvesen G. S. (2002). Caspases: opening the boxes and interpreting the arrows. *Cell Death Differ.* 9 3–5. 10.1038/sj.cdd.4400963 11803369

[B32] Santhanam A. Shihabeddin E. Atkinson J. A. Nguyen D. Lin Y. P. O’brien J. (2020). A zebrafish model of retinitis pigmentosa shows continuous degeneration and regeneration of rod photoreceptors. *Cells* 9:2242. 10.3390/cells9102242 33036185 PMC7599532

[B33] Schon C. Sothilingam V. Muhlfriedel R. Garcia Garrido M. Beck S. C. Tanimoto N. (2017). Gene therapy successfully delays degeneration in a mouse model of PDE6A-linked retinitis pigmentosa (RP 43). *Hum. Gene Ther.* 28 1180–1188. 10.1089/hum.2017.156 29212391

[B34] Sothilingam V. Garcia Garrido M. Jiao K. Buena-Atienza E. Sahaboglu A. Trifunovic D. (2015). Retinitis pigmentosa: impact of different Pde6a point mutations on the disease phenotype. *Hum. Mol. Genet.* 24 5486–5499. 10.1093/hmg/ddv275 26188004

[B35] Takahashi V. K. L. Takiuti J. T. Jauregui R. Lima L. H. Tsang S. H. (2018). Structural disease progression in PDE6-associated autosomal recessive retinitis pigmentosa. *Ophthalmic Genet.* 39 610–614. 10.1080/13816810.2018.1509354 30153077

[B36] Tsang S. H. Sharma T. (2018a). Autosomal dominant retinitis pigmentosa. *Adv. Exp. Med. Biol.* 1085 69–77. 30578488 10.1007/978-3-319-95046-4_15

[B37] Tsang S. H. Sharma T. (2018b). Retinitis pigmentosa (Non-syndromic). *Adv. Exp. Med. Biol.* 1085 125–130. 30578498 10.1007/978-3-319-95046-4_25

[B38] Tuntivanich N. Pittler S. J. Fischer A. J. Omar G. Kiupel M. Weber A. (2009). Characterization of a canine model of autosomal recessive retinitis pigmentosa due to a PDE6A mutation. *Invest. Ophthalmol. Vis. Sci.* 50 801–813. 10.1167/iovs.08-2562 18775863 PMC3720143

[B39] Ullah I. Kabir F. Gottsch C. B. Naeem M. A. Guru A. A. Ayyagari R. (2016). Mutations in phosphodiesterase 6 identified in familial cases of retinitis pigmentosa. *Hum. Genome Var.* 3:16036. 10.1038/hgv.2016.36 PMC5112436 27917291

[B40] Venkatraman P. Mills-Henry I. Padmanabhan K. R. Pascuzzi P. Hassan M. Zhang J. (2020). Rods contribute to visual behavior in larval zebrafish. *Invest. Ophthalmol. Vis. Sci.* 61:11. 10.1167/iovs.61.12.11 33049059 PMC7571310

[B41] Verbakel S. K. Van Huet R. A. C. Boon C. J. F. Den Hollander A. I. Collin R. W. J. Klaver C. C. W. (2018). Non-syndromic retinitis pigmentosa. *Prog. Retin. Eye Res.* 66 157–186. 29597005 10.1016/j.preteyeres.2018.03.005

[B42] Zelinka C. P. Sotolongo-Lopez M. Fadool J. M. (2018). Targeted disruption of the endogenous zebrafish rhodopsin locus as models of rapid rod photoreceptor degeneration. *Mol. Vis.* 24 587–602. 30210230 PMC6128699

[B43] Zhuang Y. Y. Xiang L. Wen X. R. Shen R. J. Zhao N. Zheng S. S. (2019). Slc7a14 is indispensable in zebrafish retinas. *Front. Cell Dev. Biol.* 7:333. 10.3389/fcell.2019.00333 31921845 PMC6920099

